# Inflammatory Cytokines in Diabetic Kidney Disease: Pathophysiologic and Therapeutic Implications

**DOI:** 10.3389/fmed.2020.628289

**Published:** 2021-01-22

**Authors:** Javier Donate-Correa, Carla M. Ferri, Fátima Sánchez-Quintana, Atteneri Pérez-Castro, Ainhoa González-Luis, Ernesto Martín-Núñez, Carmen Mora-Fernández, Juan F. Navarro-González

**Affiliations:** ^1^Unidad de Investigación, Hospital Universitario Nuestra Señora de Candelaria, Santa Cruz de Tenerife, Spain; ^2^GEENDIAB (Grupo Español para el Estudio de la Nefropatía Diabética), Sociedad Española de Nefrología, Santander, Spain; ^3^Doctoral and Graduate School, University of La Laguna, San Cristóbal de La Laguna, Spain; ^4^REDINREN (Red de Investigación Renal-RD16/0009/0022), Instituto de Salud Carlos III, Madrid, Spain; ^5^Instituto de Tecnologías Biomédicas, Universidad de La Laguna, Santa Cruz de Tenerife, Spain

**Keywords:** diabetic kidney disease, inflammation, inflammatory cytokines, SGLT2i, GLP-1RA, DPP-4i, pentoxifylline

## Abstract

Diabetic kidney disease (DKD) is the leading cause of end-stage renal disease and a main contributing factor for cardiovascular morbidity and mortality in patients with diabetes mellitus. Strategies employed to delay the progression of this pathology focus on the control of traditional risk factors, such as hyperglycemia, and elevated blood pressure. Although the intimate mechanisms involved in the onset and progression of DKD remain incompletely understood, inflammation is currently recognized as one of the main underlying processes. Untangling the mechanisms involved in the appearing of a harmful inflammatory response in the diabetic patient is crucial for the development of new therapeutic strategies. In this review, we focus on the inflammation-related pathogenic mechanisms involved in DKD and in the therapeutic utility of new anti-inflammatory strategies.

## Introduction

Diabetic kidney disease (DKD) is a frequent complication in patients with diabetes mellitus (DM) and constitutes the first cause of kidney disease and an important risk factor for cardiovascular disease in this population (1, 2). DKD is characterized by a plethora of alterations including hemodynamic and metabolic abnormalities, the activation of the renin–angiotensin system (RAS), oxidative stress, and fibrosis, which together trigger the elevation of systemic and intraglomerular pressure, and the appearing of various symptoms related to the development of kidney failure: glomerular hypertrophy, proteinuria, and decreased glomerular filtration (3). Until recently, kidney involvement was only considered as the result of alterations in hemodynamic and metabolic factors; however, recent advances show that DKD is a complex and multifactorial process.

An increasing number of clinical and experimental studies have pointed out that inflammation contributes to the development and progression of DKD (4). Therefore, inflammatory mediators may represent new targets for the development of therapeutic strategies for the treatment of this complication. In this review, we discuss the pathophysiologic and therapeutic implications of the inflammatory cytokines in DKD.

## Inflammatory Cytokines in The Setting of DKD

**Figure 1:**
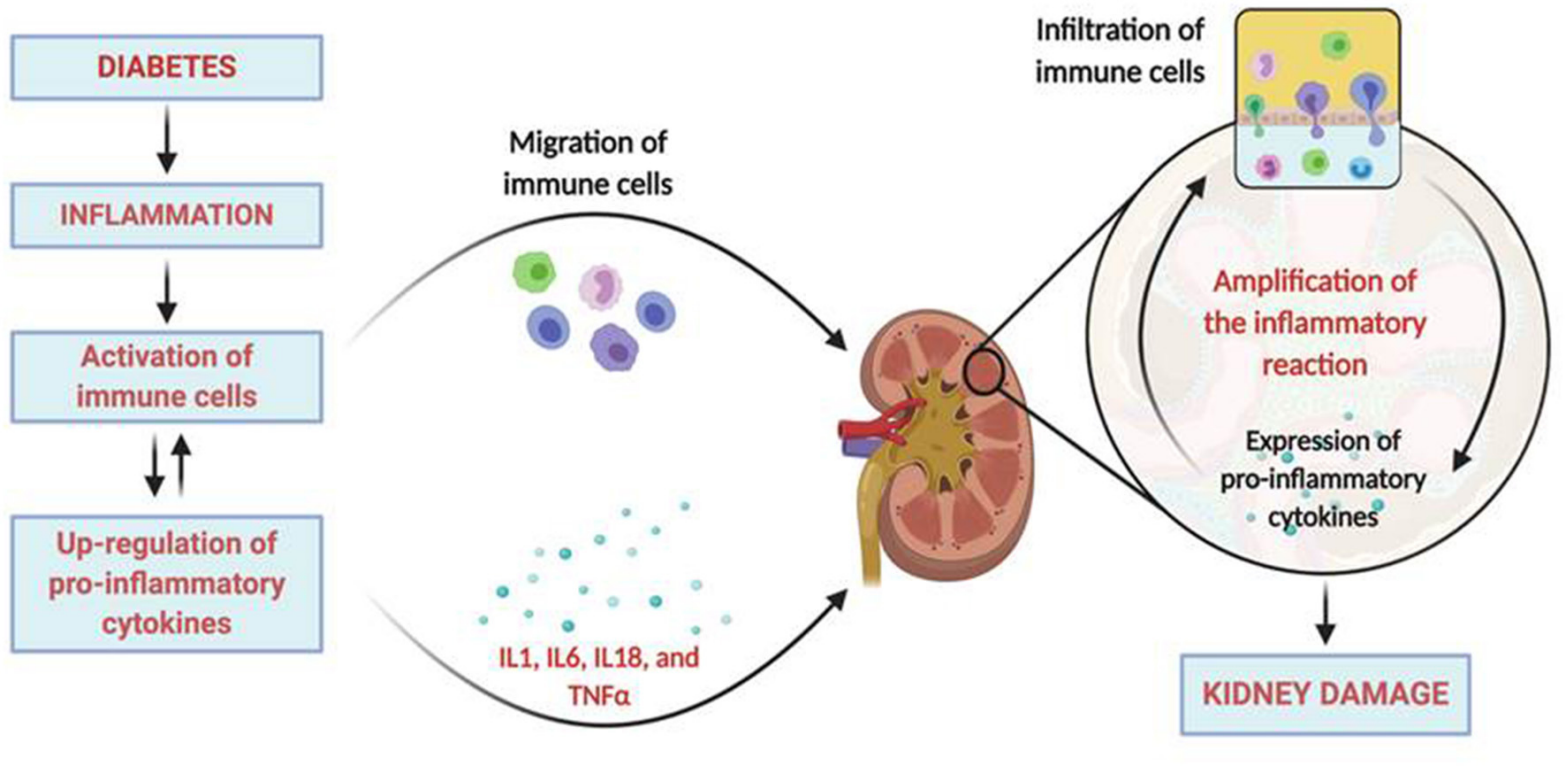
Inflammatory pathophysiology in diabetic kidney disease. The diabetic milieu drives to the development of inflammation that includes the activation of immune cells and the upregulation of pro-inflammatory cytokines. Activated immune cells migrates and infiltrates the renal tissue locally producing more inflammatory mediators and chemokines that recruit more immune cells into the kidney. Moreover, activated resident renal cells can also produce additional proinflammatory mediators, contributing to sustained inflammation, and the induction of kidney damage. Created with BioRender.com.

Diverse inflammatory parameters are predictors of the evolution of the DM (5, 6), including the initiation and progression of DKD (4). These include inflammatory cytokines which, in addition to their role as regulators of the immune response, also exert important actions as cardinal effectors of injury. The increase in the levels of these molecules in the diabetic patient leads to microvascular complications such as the development of nephropathy (7–9). Proinflammatory cytokines, both circulating and those synthesized and secreted by inflammatory cells in the renal tissue, are elevated in patients with DKD (10–13), being directly associated with urinary albumin excretion (UAE) levels and with clinical markers of glomerular and tubulointerstitial damage (14, 15) (Figure 1).

Urinary levels of interleukin (IL) 6 and IL8 are significantly increased in microalbuminuric diabetic patients with early progressive renal function decline when compared to microalbuminuric or normoalbuminuric subjects with stable renal function (16). IL6 levels are upregulated in patients with DM and nephropathy (17); in the same way, the expression of mRNA encoding IL6 is also increased in renal cells (glomerular, epithelial, mesangial), as well as in kidney infiltrating-cells in patients with DKD compared to diabetic patients without kidney disease (17). Importantly, the expression levels of IL6 mRNA are positively related with the severity of mesangial expansion, a characteristic histological manifestation of DKD.

IL18 is another potent pro-inflammatory cytokine that exhibits elevated serum and urinary levels in patients with DKD, showing significant and direct correlations with UAE levels and with the evolution of albuminuria (7, 18, 19). Additionally, serum levels of IL18 are associated with urinary β-2 microglobulin, a marker of tubular dysfunction (18). IL18 is capable of regulating the synthesis of pro-inflammatory molecules such as IL1 and tumor necrosis factor (TNF) α, and interferon (IFN) ɤ (20), in turn increasing chemokine receptors in mesangial cells (21). Furthermore, IL8 increases the expression of intercellular adhesion molecule 1 (ICAM-1) (22) and promotes endothelial cell apoptosis (23). In addition to infiltrating cells, kidney tubular cells of patients with DKD also express increased levels of IL18 (24), which is related to the activation of the mitogen-activated protein kinase (MAPK) pathways by transforming growth factor (TGF) β (25).

Both serum and urinary levels of the cytokine TNFα are also elevated in patients with DKD (26). Moreover, this increase parallels the progression of renal damage, which may indicate a relationship with the development and progression of renal impairment in the diabetic patient (7, 9, 27). In these subjects, the rise in TNFα has been related with renal damage mediated by cytotoxic effects (26). Similar to IL18, TNFα is also expressed in endothelial, epithelial, mesangial, and tubular renal cells (28, 29). Importantly, animal models of diabetes present increased levels of TNFα in kidney glomeruli and tubules (9, 30–33) being directly and independently related with UAE (32).

Animal models of DKD show upregulation of IL1 expression in many types of kidney cells (31, 34). This increment has been related with the expression of ICAM-1, the vascular cell adhesion molecule-1 (VCAM-1), and the endothelial-leukocyte adhesion molecule-1 (ELAM-1) (35, 36), molecules involved in chemotaxis and adhesion processes. Rat kidney mesangial cells are stimulated to produce prostaglandin E2 after being incubated with recombinant IL1 (37) in response to Ang II (38), which is related to the appearing of abnormalities in intraglomerular hemodynamics. Finally, IL1 has been also related with the production of hyaluronan in the proximal tubule (39), which is related with the development of experimental hypercellularity (40).

The cytokine IL17A is a member of the IL17 family mainly produced by activated T helper (Th) lymphocytes Th17 and, at lesser extent, by macrophages, neutrophils, natural killer, dendritic, and mast cells. Circulating IL17A levels have been related with the severity of kidney disease, showing a progressive decrease from subjects with normal glucose tolerance to subjects with DM with and without DN (41, 42). In opposite with these findings, other authors have found increased IL17A plasma values in patients with DN compared to healthy controls (43). These paradoxical findings might by the fact that the modulatory effects of IL17A on inflammation may be dependent on disease context, tissue, isoform, and receptor-ligand interactions (44).

## Pathophysiologic Implications

The pathophysiological implications of the inflammation on DKD occur at various levels. In a first level, inflammatory cytokines, acting in a paracrine or autocrine form, trigger the epithelial-to-mesenchymal transition process in the kidney (45), causing extracellular matrix accumulation. Secondly, the up-regulation of chemoattractant cytokines and adhesion molecules stimulates the attraction of circulating cells and facilitate their infiltration into the renal tissue. Finally, these cells amplify the inflammatory reaction, generating more cytokines and other mediators that contribute to the development and progression of renal injury (Figure 1).

The first clue suggesting the existence of a pathogenic role of these molecules in the DKD was obtained *in vitro* by incubating macrophages in a glomerular basement membrane derived from diabetic rats; these macrophages presented increased production of IL1 and TNFα when compared to macrophages incubated with membranes from normal animals (8).

The interleukins IL1, IL6, and IL8, have been clearly related to renal disorders in DKD (31, 34). IL1 has been linked with the proliferation of mesangial cells and matrix synthesis, thereby altering the renal architecture, with increased permeability of vascular endothelial cells, and with intraglomerular hemodynamic abnormalities secondary to alterations in prostaglandin production (37, 46). In DKD patients, the expression of mRNA molecules encoding *IL6* is positively related with the severity of mesangial expansion (17). The up-regulation of this cytokine have been also related to another functional and structural abnormalities related to DKD including increased vascular endothelium permeability (46), mesangial expansion and fibronectin expression (47), thickening of the glomerular basement membrane (48, 49), and renal hypertrophy (50). High serum and urine levels of the IL18 in type 2 diabetes have been proposed as early predictors of renal dysfunction, being related with the appearing of macroalbuminuria (18) and with the triggering of MAPK pathways by TGF-β (24). High serum TNFα levels have been clearly related with the pathophysiology of DKD including the development of renal hypertrophy and hyperfiltration (33). Levels of sodium retention, which is present in the early stages of DKD, has been related with urinary TNFα (51). Sodium retention in turn induce the expression of TGFβ and the development of renal hypertrophy (52). Moreover, the rise in both urinary and kidney-expressed TNFα levels precede the rise in albuminuria (53). These harmful effects elicited by TNFα in the kidney are mediated by the cytotoxicity on glomerular, mesangial, and epithelial cells (54–57). TNFα also change the permeability of endothelial cells, altering the equilibrium between vasoconstriction and vasodilation and the intraglomerular blood flow, which reduces glomerular filtration rate (GFR) independently from alterations in hemodynamic factors (58, 59). Moreover, TNFα also increases ROS levels in kidney cells independently of hemodynamic mechanisms, altering the glomerular capillary wall and, consequently, increasing UAE (51, 59, 60).

IL17A exerts mainly proinflammatory responses via the activation of the NF-κB pathway and downstream regulation of proinflammatory genes (61). In podocytes *in vitro*, IL17A increased the expression of IL 6 and TNFα, particularly under conditions of high glucose concentration (62). Similarly, cultured tubular epithelial cells stimulated with this cytokine also present up-regulation of proinflammatory gene expression and monocyte chemoattractant protein 1 (MCP1) production (62, 63). Importantly, IL17A also induced epithelial-to-mesenchymal transition in cultured proximal tubular epithelial cells (64), indicating that this could be another mechanism of renal damage triggered by this cytokine. In experimental models, administration of IL17A in mice significantly upregulated kidney Mcp-1 and Rantes gene expression and the recruitment of inflammatory cells to the kidney (65). In leptin deficient BTBR ob/ob mice, the administration of a neutralizing anti-IL17A antibody resulted in an inhibition of NF-κB activation (66). Similarly, neutralization of IL17A also decreased proinflammatory genes and inflammatory cell infiltration in an experimental angiotensin II-induced renal damage model (67). Taken together, these observations suggest that local IL17A production in diabetic kidneys could activate resident renal cells to produce proinflammatory cytokines and chemokines, such as MCP-1, thereby amplifying the inflammatory response. Although many studies have addressed circulating or urinary IL17A levels in DN patients, the determination of local renal levels of IL17A has not been investigated yet.

## Inflammation As A Therapeutic Target in DKD

Up to the present time, the main therapeutic strategy to slow the development and progression of DKD focus on the tight regulation of glucose levels and blood pressure by the utilization of RAS blockers: angiotensin converting enzyme inhibitors (ACEi) and angiotensin II receptor blockers (ARBs). Although this therapeutic approximation reduces the risk of nephropathy progression, they do not completely halt the evolution toward end-stage renal disease (ESRD), with a still significant residual renal risk.

The important role played by inflammation, and specifically the involvement of inflammatory cytokines in the pathophysiology of ERD, constitute a new therapeutic opportunity in DKD to improve kidney function by the pharmacological reduction of the levels of these molecules (Table 1). Interestingly, the reduction in proteinuria and the slowdown in the progression of diabetic and non-diabetic nephropathies derived from RAS blockade has been related not only to hemodynamic/antihypertensive but also to anti-inflammatory/antifibrotic effects. This anti-inflammatory effect is mediated by the inhibition of NF-κB dependent pathways (68), which include the production of the pro-inflammatory cytokines IL6 and TNFα (69).

**Table 1:** Therapeutic strategies in the DKD with potential anti-inflammatory properties.

**Drug**	**Primary target**	**Main outcomes in DKD**	**Anti-inflammatory effects**	**References**
RAS blockers	Inhibition of ACE or blockade of angiotensin II receptor.	Reduce proteinuria and the progression of nephropathy.	Inhibition of NF-κB, MCP1 gene expression, and macrophage infiltration.	(68, 69)
SGLT2 inhibitors	Blockade of glucose reabsorption by SGLT2 at the proximal tubule.	Improved glycemic control. Slower progression of kidney disease and lower rates of clinically relevant renal events.	Reduction of inflammation by targeting the IL1ß and reduction of hsCRP, TNFα, IL6, and IFNγ.	(70–90)
DPP4 inhibitors and GLP-1 receptor agonists	Stimulation of glucose-dependent insulin release.	Improved glycemic control and body weight reductions. Renoprotective actions	Reduction in levels of inflammatory markers including CRP, TNFα, IL6, and IL18.	(91–97)
Pentoxifylline	Inhibition of phosphodiesterases.	Reduced progression of renal disease and proteinuria.	Downregulation of NF-κB signaling and reduction of inflammatory biomarkers.	(98–114)

*RAAS, Renin-Angiotensin Aldosterone System; ACEI, angiotensin converting enzyme; NF-κB, nuclear factor-κB; MCP1, monocyte chemoattractant protein 1; SGLT2, type 2 sodium-glucose cotransporter; IL, interleukin; hsCRP, high sensitivity C reactive protein; TNFα, tumor necrosis factor α; IFN-γ, interferon γ; DPP4, dipeptidyl-peptidase-4; GLP-1, glucagon-like peptide-1*.

In recent years, new antidiabetic drugs have emerged (Table 1). These drugs include SGLT2i (sodium-glucose cotransporter type 2 inhibitors), GLP-1RA (glucagon-like peptide-1 receptor agonist), and DPP-4i (dipeptidyl peptidase-4 inhibitors). These compounds improve albuminuria and other traits of DKD in diabetic patients but also have been demonstrated to exert anti-inflammatory effects with potential benefits in the delay of the progression of DKD. The group of SGLT2i (canagliflozin, dapagliflozin, and empagliflozin, among others) are very effective hypoglycemic agents that, beyond glycemic control, have shown potent cardiovascular and renal protective effects in the diabetic patient (70–72). The underlying mechanisms of these effects are not fully understood, but recent findings suggest that are related with the modulation of inflammatory cytokines at renal and systemic levels. A few small clinical pilot studies have shown reductions of inflammatory markers in type 2 diabetes patients treated with SGLT2i. In this sense, mildly reductions in serum concentrations of C-reactive protein (CRP), TNFα, IL6, IL1β, and IFN-γ were observed in diabetic patients treated with canagliflozin, dapagliflozin, and empagliflozin (74–80). While robust data on short and mainly long-term effects of SGLT2i are obtained from clinical studies with a larger number of patients, most of what is known about the anti-inflammatory actions of these drugs comes from experimental approximations, which have made possible to delve into the underlying mechanisms of this beneficial immunomodulatory effect. In HK2 cells, a line of human kidney proximal tubule cells, empagliflozin attenuated the expression of Toll-like receptor-4 (TLR4) induced by high glucose, NF-κB and activator protein1 (AP1) transcription factors binding to nuclear DNA, and secretion of collagen IV and IL6 (81). In vascular endothelial cells, canaglifozin inhibited IL1β-stimulated IL6 and MCP1 secretion by AMP-activated protein kinase dependent and independent mechanisms (82). In obese diabetic or prediabetic animals, SGLT2i reduces albuminuria and tubulointerstitial injury (83–85). Thereby, the slowing in the progression of renal complications in prediabetic rats treated with dapagliflozin was related with the suppression of renal inflammation (84). The expression of TNFα, IL1β, and IL6, as well as the infiltration of cells into atheromatous plaques, were reduced in the atherosclerosis mice model ApoE ^−/−^ after treatment with empagliflozin (86). Empagliflozin achieved significant reductions in albuminuria in animal models with type 1 diabetes that were related with lower levels of renal NF-κB, MCP1, and IL6 (87). The inhibition of SGLT2 in experimental type 2 diabetes models, reduced glomerular macrophage infiltration and sclerosis (88) and attenuated the overexpression of NOX4, TGFβ, osteopontin, and MCP1 in the tubular cells induced by high glucose (89).

Several clinical trials suggest that the anti-diabetic drugs DPP-4i and GLP-1RA also possess beneficial renal effects (90, 91), potentially modulating the inflammatory response. The DPP-4i vildagliptin and sitagliptin reduced the levels of markers of inflammation in diabetic patients (92). Administration of GLP-1RA, exenatide or dulaglutide, reduced the levels of CRP in diabetic patients (93). Different clinical trials are presently evaluating the effects of these anti-diabetic therapeutic agents on systemic and renal inflammation: GLP-1RA (exenatide or liraglutide) *vs*. a DPP-4i (NCT02150707) and liraglutide (LIRALBU; NCT02545738 and NCT01847313). In the meantime, the renoprotective effects derived from the treatment with GLP-1RA and DPP-4i have been demonstrated in the experimental setting employing animal models with kidney disease. The DPP-4i linagliptin reduced albuminuria, glomerulosclerosis, and tubulointerstitial fibrosis, independently of the glycemic control, in a mouse model of DKD (94). Sitagliptin and linagliptin also suppressed the activity of NLRP3/inflammasome in an experimental model of nephropathy (95). The model of type 2 diabetes db/db mice reduced albuminuria levels and mesangial expansion after treatment with the GLP-1RA exenatide (96). This treatment also reduced the levels of inflammatory cytokines and adhesion molecules in type 1 in diabetic rats (97).

The utilization of microRNAs (miRNAs) to regulate Th17 cell differentiation by targeting transcription factors like orphan receptor γt (RORγt) and the activation of STAT-3 or key cytokines involved in this process have been tested in different models of autoimmune disease (115–118). In the context of kidney disease, a recent study described that miRNA-155 deficiency promoted nephrin acetylation and decreased renal damage in a hyperglycemia-induced nephropathy mice model; importantly these effects were associated with a reduction in IL17A production through the up-regulation of the suppressor of cytokine signaling 1 (SOCS1) expression (119). Similarly, miR-155 knockout mice presented a significant reduction of the Th17 immune response, less severe nephritis, and reduced histologic and functional injury in an experimental model of nephritis (120).

Another potential therapeutic opportunity is the administration of neutralizing antibodies against IL17A for the treatment of chronic human inflammatory diseases is being tested in several ongoing clinical trials (121, 122). Anti-IL17A biological agents like secukinumab, ixekizumab, and brodalumab were more effective than diverse anti-TNF alpha agents such as infliximab, adalimumab, and etanercept, but less so than certolizumab. Unfortunately, although it seems clear that IL17A could play an important role in the inflammatory component of kidney disease, there are no clinical trials focused on the effects of anti-IL17A antibodies on renal inflammatory diseases like DKD or even lupus nephritis (123).

Pentoxifylline (PTX) is a methyl-xanthine clinically used for the treatment of intermittent claudication with anti-inflammatory properties. Both experimental and clinical studies in diabetic patients support the renal protective action of PTX, evidenced by a decrease in proteinuria and, in some cases, an improvement in GFR (98–101, 108). It is important to note that this antiproteinuric capacity is related to an anti-inflammatory effect (102–107), being associated with significant reductions in TNFα levels (108, 109). Similarly, CKD patients report a reduction in TNFα, fibrinogen, and CRP levels and a stabilization in kidney function after PTX treatment (110). In these same patients, 1-year treatment of PTX in addition to the ARB background therapy resulted in a reduction in proteinuria and urinary levels of TNFα and MCP1 (111). In the PREDIAN trial, DKD patients under RAS blockade and treated with PTX also presented a stabilization in the progression of kidney disease after 2 years of follow-up (112). Importantly, this outcome was accompanied by reductions in proteinuria and urinary TNFα levels. Two recent meta-analysis in patients with DKD indicate, on the one hand, that the antiproteinuric effect of PTX in these patients is due to a lower production of pro-inflammatory cytokines (113) and, on the other hand, that PTX additively reduces both proteinuria and TNFα levels in patients treated with RAS inhibitors (114).

## Discussion

The dramatic increase in the incidence of diabetes has prompted new therapeutic strategies to treat DKD. Inflammation has revealed as a key factor in the development and progression of this complication, allowing the development of therapeutic approaches focused on the modulation of inflammatory processes. However, at the present time, clinical experience on the inhibition of inflammatory molecules and pathways in diabetic patients is scarce and more clinical trials are needed to examine their potential renoprotective efficacy.

## Author Contributions

JN-G, JD-C, and CF conceived the idea, prepared the final version, and finalized the manuscript. CF searched the literature, prepared the first and last part of the draft, and reviewed and prepared the revised version. FS-Q prepared the third part of the draft. AP-C, AG-L, EM-N, and CM-F reviewed the literature, critically reviewed the final version, and approved the final manuscript. All authors have substantially contributed to the preparation of the manuscript and agree with the study.

## Conflict of Interest

The authors declare that the research was conducted in the absence of any commercial or financial relationships that could be construed as a potential conflict of interest.
